# Supplementary material for: Structures of ISC th4 transpososomes reveal the role of asymmetry in copy‐out/paste‐in DNA transposition
Source: EMBO J. 2020 Oct 2;40(1):e105666. doi: 10.15252/embj.2020105666 (PMC7780238; doi:10.15252/embj.2020105666)
Supplement: Supplementary file 1 — Appendix [file EMBJ-40-e105666-s001.pdf]

Index of Appendix Figures

1. Appendix Figure S1: Purification of TnpA, biochemical characterization of active site mutant D175A and SDS-PAGE verification of PCC crystal

2. Appendix Figure S2: Sequence-based alignment of transposases of IS*Cth4* and IS*256*

3. Appendix Figure S3: Analysis of TnpA oligomerization

4. Appendix Figure S4: AUC analysis of PCC complex, asymmetry of PCC on molecular level

5. Appendix Figure S5: Comparison of known α-helical insertion domains across DDE transposases, superposition of PCC active center with Hermes

6. Appendix Figure S6: Integration of minimal asymmetric TIR junction

7. Appendix Figure S7: Representative composite simulated annealed omit maps in PCC, close-up views of active sites in PRC, PCC and STC1

8. Appendix Table S1: List of oligonucleotides and DNA fragments used throughout the study

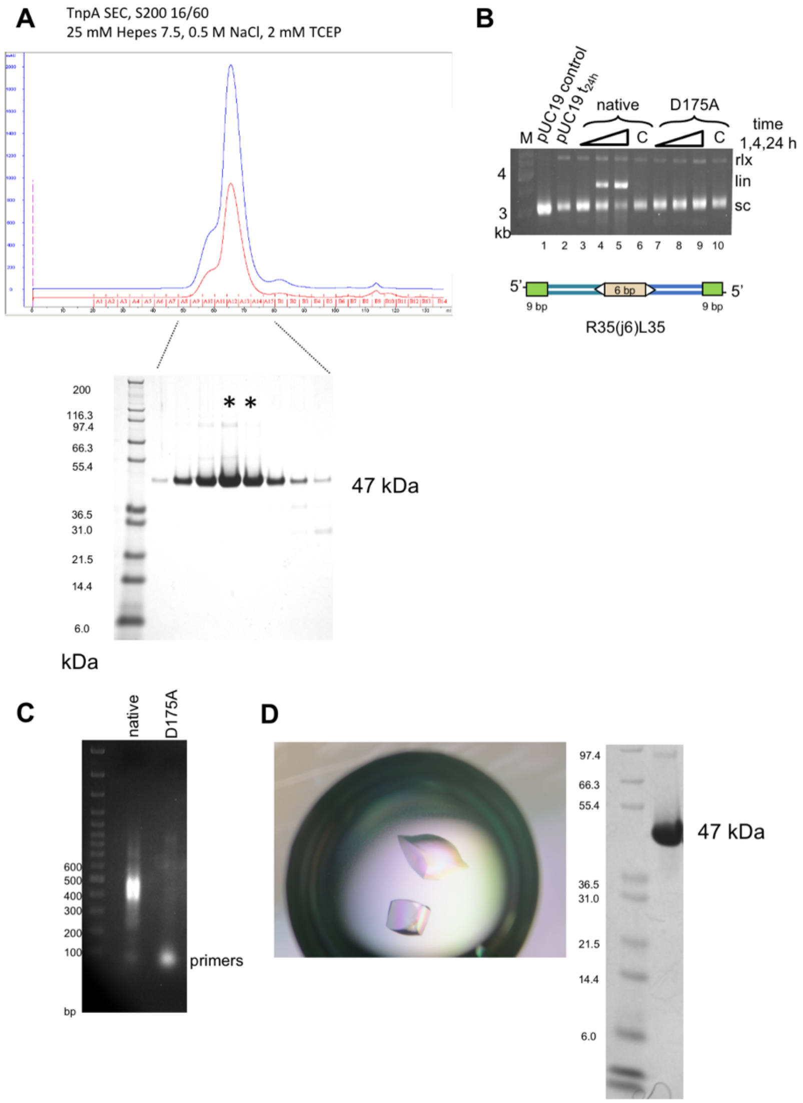

Appendix Figure S1 – Purification of TnpA, biochemical characterization of active site mutant D175A, and SDS-PAGE verification of PCC crystal

A (top) Size-exclusion chromatography of IS*Cth4* TnpA on a Superdex 200 16/60 column run in 25 mM HEPES pH 7.5, 0.5 M NaCl, and 2 mM TCEP as a last purification step. (bottom) SDS-PAGE analysis of the fractions with indicated M_w standards. Fractions marked with asterisks were combined for further experiments.

B *In vitro* integration of R35(j6)L35 junction mimic, comparison of native TnpA (lanes 3-6) and TnpA with active site D175A mutation (lanes 7-9). Lane 1, pUC19 alone at t = 0. Lane 2, pUC19 after incubation for 24 h in reaction buffer missing the TIR oligonucleotide and TnpA. Lanes 6 and 10 (“C”), pUC19 after incubation for 24 h in reaction buffer omitting the TIR oligonucleotide but with TnpA as indicated.

C *In vitro* Figure 8 detection with PCR, comparison of native TnpA and TnpA with D175A mutation.

D PCC crystals and their analysis by SDS-PAGE. M_w standards are indicated.

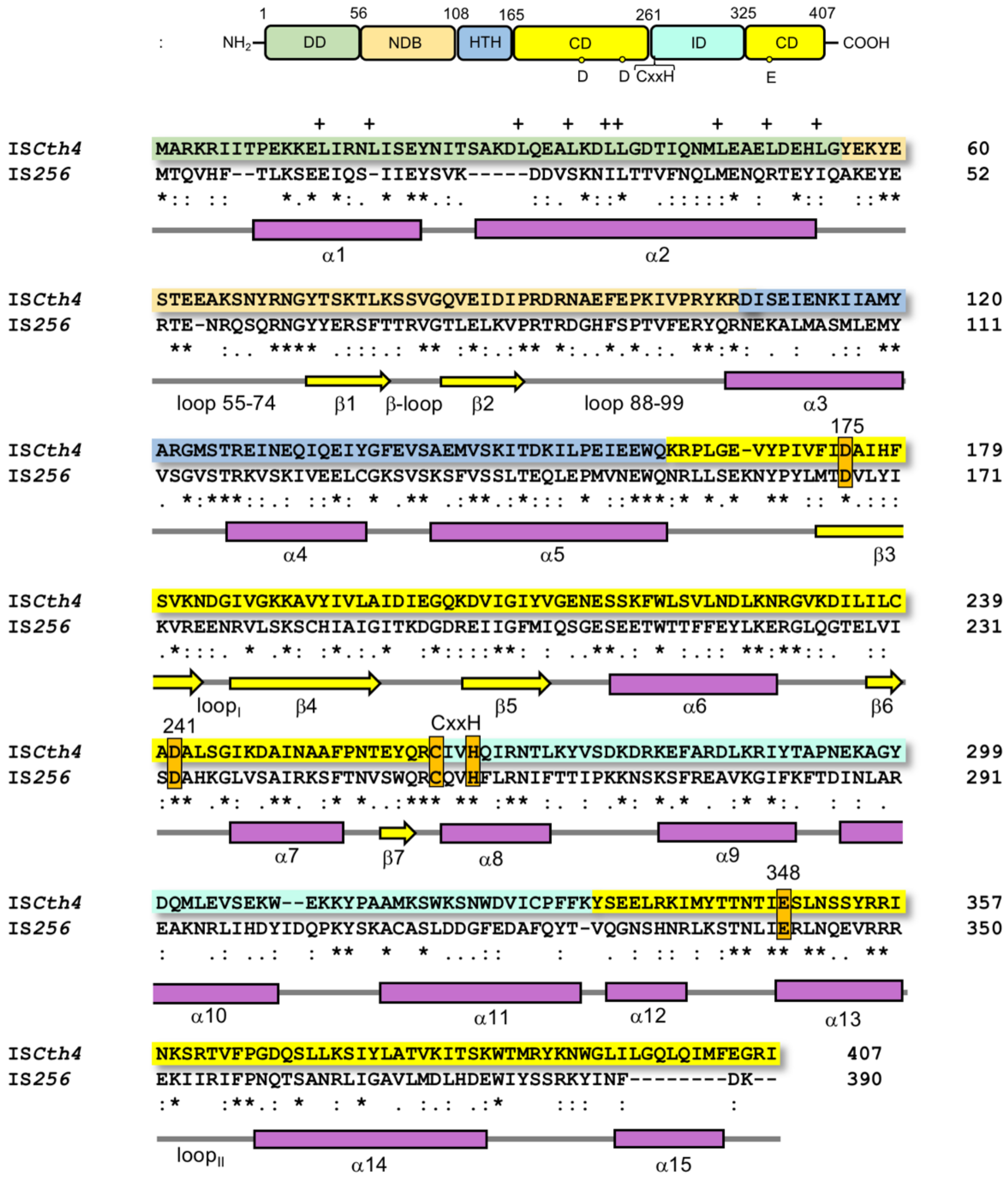

Appendix Figure S2 - Sequence-based alignment of transposases of IS*Cth4* and IS*256*

Sequence-based alignment of IS*Cth4* and IS*256* transposases. Secondary structure elements for IS*Cth4* TnpA are depicted as seen in the PCC. The conserved active site DDE and CxxH residues are highlighted in orange boxes. Leucine residues in the DD are marked with plus signs. DD, dimerization domain. NDB, N-terminal DNA-binding domain. HTH, helix-turn-helix domain. CD, catalytic domain. ID, insertion domain.

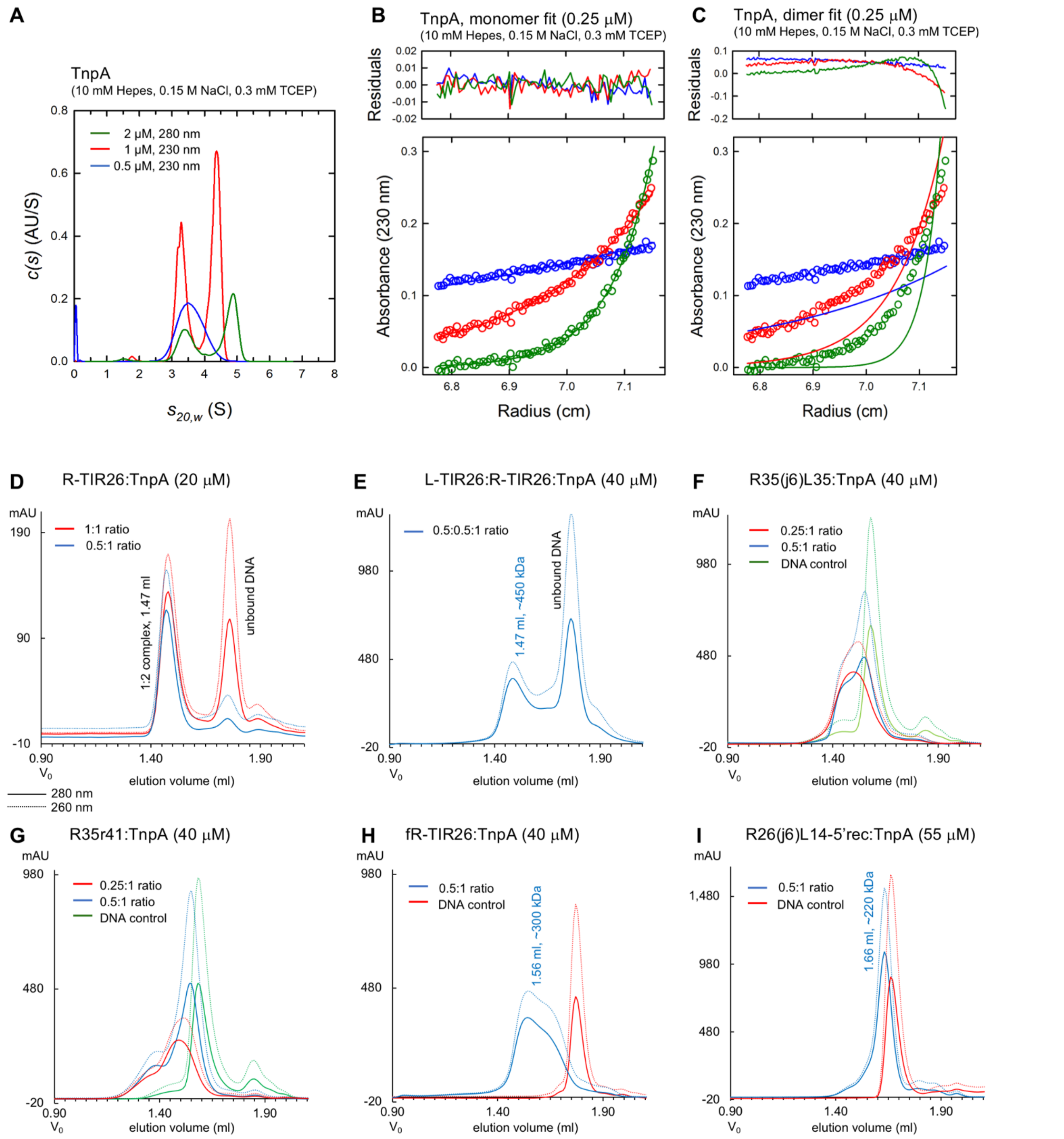

Appendix Figure S3 - Analysis of TnpA oligomerization

A SV AUC analysis of TnpA alone in the 0.5-2 μM concentration range.

B,C SE AUC analysis of TnpA alone at 0.25 μM. Data were analyzed in terms of a single ideal solute with mass conservation.3

D,E,F,G,H,I SEC analysis of TnpA mixed with various DNA substrates, ratios and concentrations indicated in panels. The assay was used as a guide for identification of monodisperse samples for crystallography. The elution positions of M_W standards are as follows: (blue dextran) V_0 = 0.9 ml, 660 kDa =1.40 ml, 440 kDa =1.55 ml, 200 kDa =1.65 ml

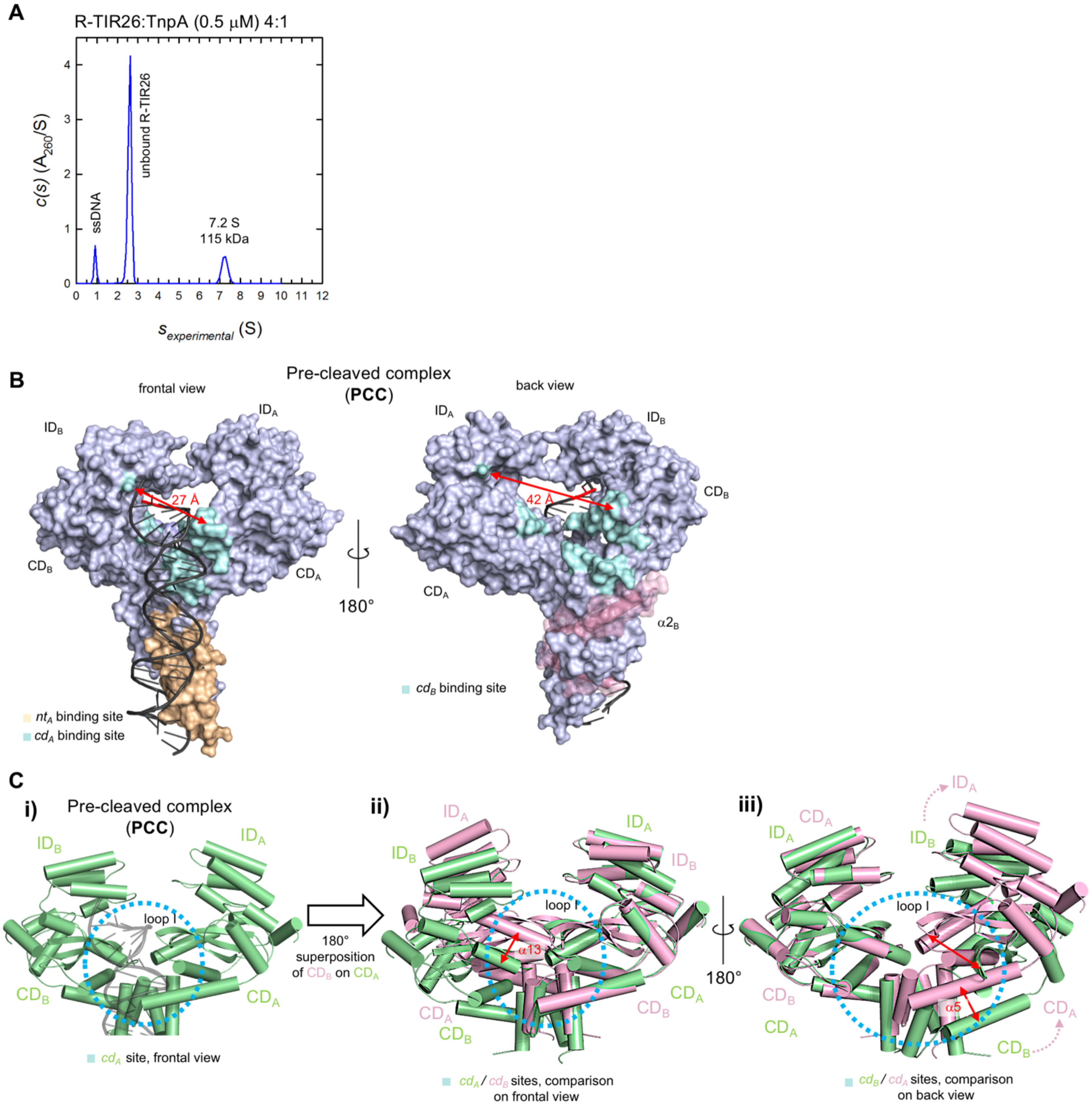

Appendix Figure S4 - AUC analysis of PCC complex and asymmetry of the PCC on the molecular level

A AUC SV analysis of the complex between R-TIR26 and TnpA with M_w estimation.

B Surface represention of PCC from front and back side. Red arrows shows distances between Cα of V185 in loop I of CD_A and K284 in CD_B (and vice versa on the back view). The change in distance illustrates the dramatic difference in conformation between binding sites cd_A and cd_B in the PCC.

C Asymmetry of catalytic domain positions in PCC and its effect on cd_A and cd_B binding sites. (i) Position of CD_A+ID_A and CD_B+ID_B from the front side (in green). cd_A is indicated by the blue circle, and R-TIR is semi-transparent for clarity. (ii) When CD_A and CD_B are rotated together about 180° (in pink) CD_B can be superposed on original non-rotated CD_A (green) up to ~1 Å rmsd, but the resulting position of

their counterparts differs; for example, note the shift in position of α13 (indicated by red arrow). R-TIR was removed for clarity. (iii) The same superposition as (ii) but rotated about 180°; the blue circle marks cd_B on the original back side (green). Notable shifts such as the position of loop I and α5 are indicated by red arrows. Changes in ID and CD are also indicated showing changes resulting in the absence of a symetrically bound second DNA molecule.

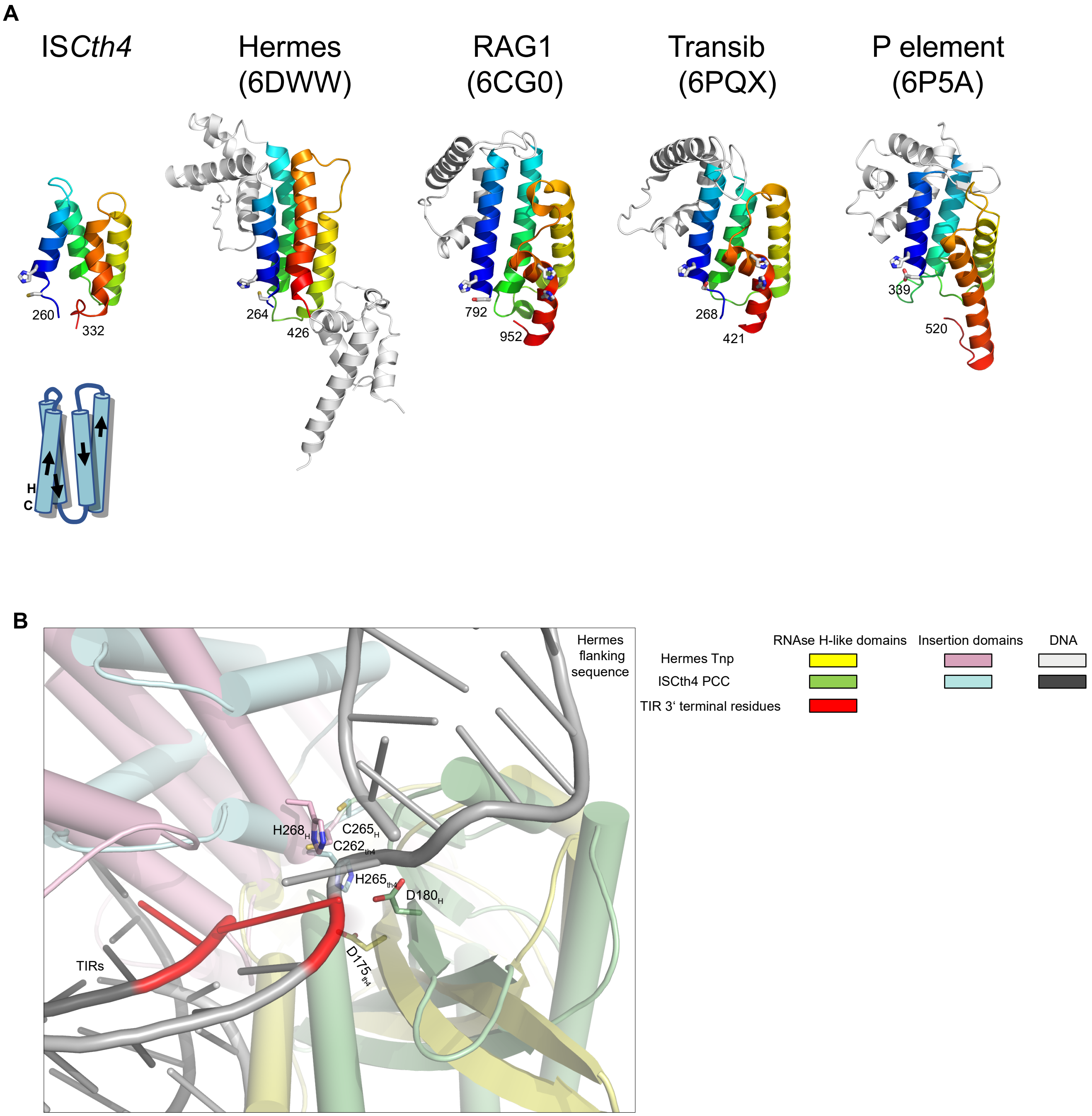

Appendix Figure S5 - Comparison of α-helical insertion domains across structurally characterized DDE transposases and active site superposition of PCC with Hermes

A Structure-based alignment of transposase insertion domains based on their common four α-helix cores which are highlighted in rainbow colors from N to C, with residue numbers shown. Insertions into the common core are shown in white. In the case of Transib and RAG1, after the last strand of the RNase H domain, the core insertion domain is preceded by a small region (not shown) that contributes two Cys ligands to a bound Zn ion (Liu et al, 2019, Ru et al, 2018). In Hermes, the core insertion domain is followed by another ~130 residues (shown) which is involved in multimerization (Hickman et al, 2014).

B Superposition of catalytic domains of IS*Cth4* Tnp (PCC) and Hermes Tnp (pdb code 6DX0). Residues of the CxxH motif for both proteins (marked in subscript H for Hermes or th4 for IS*Cth4* Tnp) are shown as sticks.

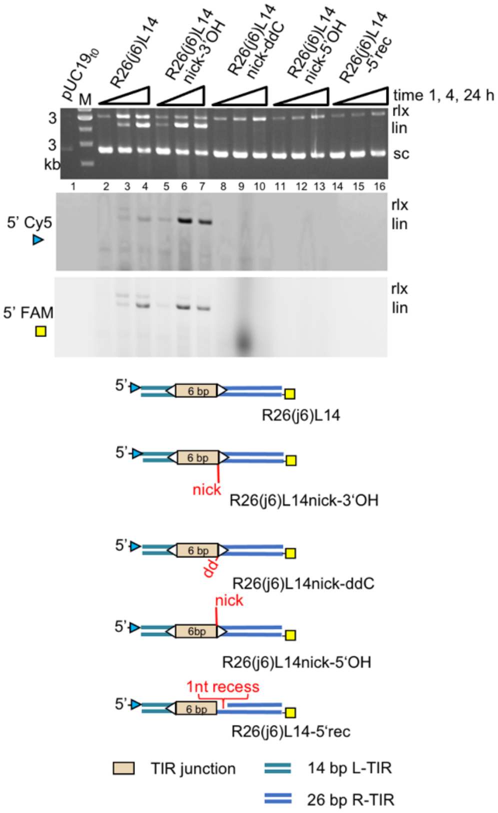

Appendix Figure S6 – Integration of minimal asymmetric TIR junctions

In vitro integration of minimal asymmetric TIR junction mimics with variations at the R-TIR or spacer boundary.

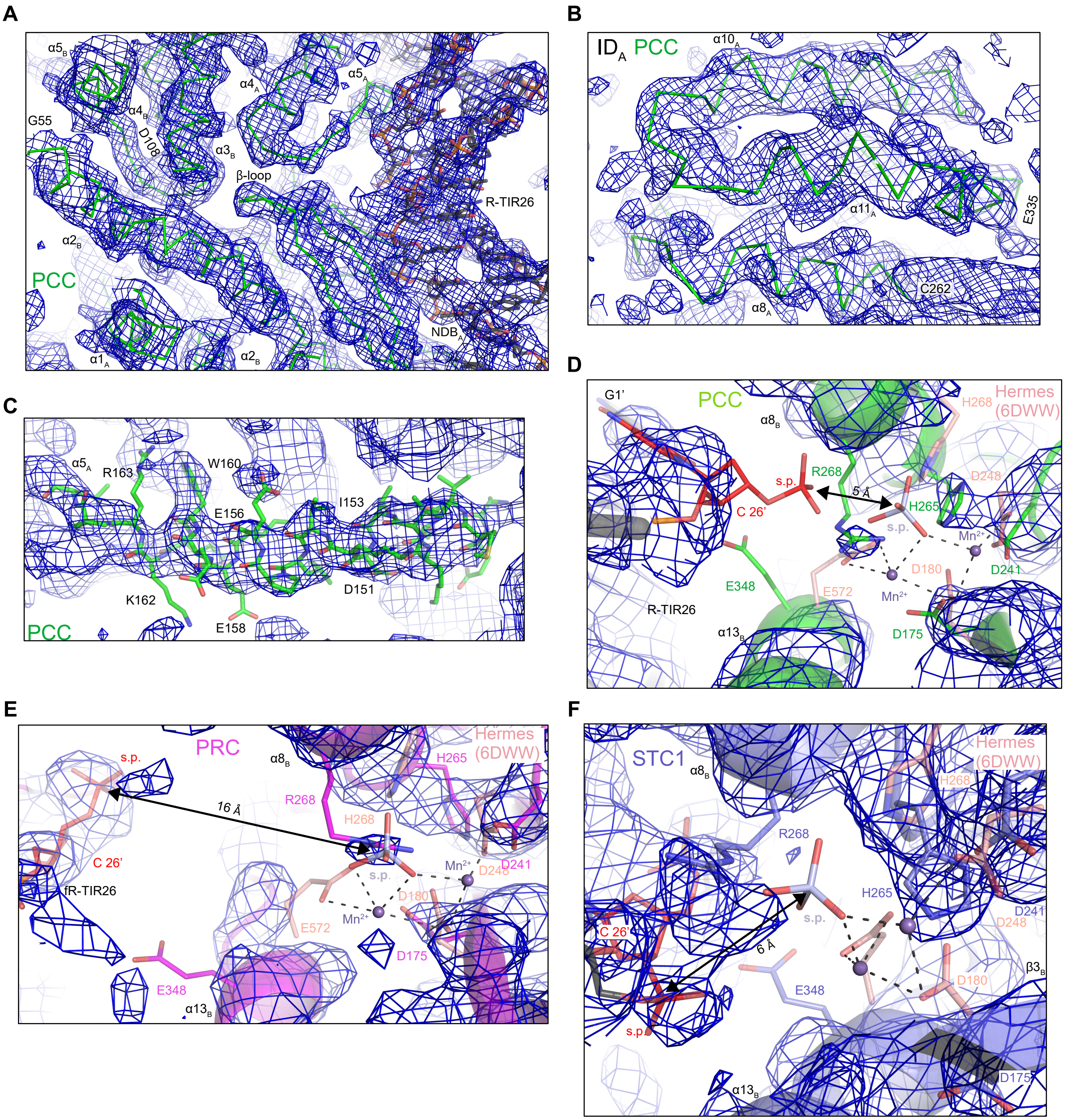

Appendix Figure S7 – Representative composite simulated annealed omit maps in PCC and close-up views of PRC, PCC and STC1 active sites

A Representative composite simulated annealed omit map of the array of N-terminal domains bound to R-TIR in the PCC (σ = 1.0).

B Representative composite simulated annealed omit map of ID in protomer A of the PCC (σ = 1.0).

C Representative composite simulated annealed omit map of α5 of protomer A (residues 143-165) in the PCC (σ = 1.0).

D Overlay of active sites of PCC and Hermes complex (6DWW; Hickman et al, 2018). PCC in green, stick representation of Hermes active site residues in pink. Scissile phosphate (s.p.) in Hermes structure in light blue. Scissile phosphate (s.p.) at the 3'-OH of R-TIR (in red) was modeled based on ideal geometry but it is not present in the PCC crystal structure. Black dashed lines indicate metal binding geometry in the Hermes complex. Black arrow indicates the distance between the positions of the scissile phosphates in the two structures. The PCC electron density map ($2mF_o$-Df_c in blue) is presented at $\sigma = 1.0$.

E Overlay of active sites of PRC and Hermes complex (6DWW). PRC in magenta, stick representation of Hermes active center residues in pink. Scissile phosphate (s.p.) in fR-TIR26 indicated in red, that in Hermes structure in light blue. Black dashed lines shows metal binding geometry in Hermes complex. The PRC electron density map ($2mF_o$-Df_c) in blue at $\sigma = 1.0$.

F Overlay of active sites of STC1 and Hermes complex (6DWW). STC1 in blue, stick representation of Hermes active center residues in pink. Scissile phosphate (s.p.) in R26(j6)L15-5'rec (s.p.) indicated in red, that in Hermes structure in light blue. Black dashed lines shows metal binding geometry in Hermes complex. Black arrow indicates the distance between equivalent oxygen atoms in scissile phosphates in both structures. The STC1 electron density map ($2mF_o$-Df_c) in blue at $\sigma = 1.0$.

Appendix Table S1 – List of oligonucleotides and DNA fragments used throughout the study

Primers (IDT)

primer 1

5'-cgtataagcttaataattttgtttaactttaagaaggagatataatgagcgataaaattattcacctg

primer 2

5'-agtacgggtaccttattaaatgcggccttcaaacataat

primer 3

5'-ggcagtgtaaatatttttgtgtaaactaattgactcaaaatcccttaacgtgagttttcgttcca

primer 4

5'-catgagattatcaaaaaggatcttcaccta

det1

5'-cattaggcaccccaggcttt

det2

5'-gctgaagccagttaccttcggaa

ssDNA for oligonucleotides used in the study (IDT)

R-TIR10

5'-ggcagtgtaa

5'-ttacactgcc

R-TIR15

5'-ggcagtgtaaatatt

5'-aatatttacactgcc

R-TIR20

5'-ggcagtgtaaatatttttgt

5'-acaaaaatatttacactgcc

R-TIR25

5'-ggcagtgtaaatatttttgtgtaaa

5'-tttacacaaaaatatttacactgcc

R-TIR26

5'-ggcagtgtaaatatttttgtgtaaac

5'-gtttacacaaaaatatttacactgcc

R-TIR27

5'-ggcagtgtaaatatttttgtgtaaact

5'-agtttacacaaaaatatttacactgcc

R-TIR30

5'-ggcagtgtaaatatttttgtgtaaactaat

5'-attagtttacacaaaaatatttacactgcc

R-TIR35

5'-ggcagtgtaaatatttttgtgtaaactaattttcc

5'-ggaaaattagtttacacaaaaatatttacactgcc

random26 mer labeled

5'FAM-cgtactgcatgcatgcaactgcacga

5'-tcgtgcagttgcatgcatgcagtacg

r14R12

5'-acttcagctacgtgttttgtgtaaac

5'-gtttacacaaaacacgtagctgaagt

R35(j0)L35

5'-gcactggatgtttacacaaaaatatttacactgccgagattgtaaaatattttatgtaaacagtgcactg

5'-cagtgcactgtttacataaaatattttacaatctcggcagtgtaaatatttttgtgtaaacatccagtgc

R35(j2)L35

5'-gcactggatgtttacacaaaaatatttacactgccttgagattgtaaaatattttatgtaaacagtgcactg

5'-cagtgcactgtttacataaaatattttacaatctcaaggcagtgtaaatatttttgtgtaaacatccagtgc

R35(j4)L35

5'-gcactggatgtttacacaaaaatatttacactgccttttgagattgtaaaatattttatgtaaacagtgcactg

5'-cagtgcactgtttacataaaatattttacaatctcaaaaggcagtgtaaatatttttgtgtaaacatccagtgc

R35(j5)L35

5’-gcactggatgtttacacaaaaatatttacactgcctttttgagattgtaaaatattttatgtaaacagtgcactg

5’-cagtgcactgtttacataaaatattttacaatctcaaaaaggcagtgtaaatatttttgtgtaaacatccagtgc

R35(j6)L35

5’-gcactggatgtttacacaaaaatatttacactgccttttttgagattgtaaaatattttatgtaaacagtgcactg

5’-cagtgcactgtttacataaaatattttacaatctcaaaaaaggcagtgtaaatatttttgtgtaaacatccagtgc

R35(j7)L35

5’-gcactggatgtttacacaaaaatatttacactgcctttttttgagattgtaaaatattttatgtaaacagtgcactg

5’-cagtgcactgtttacataaaatattttacaatctcaaaaaaaggcagtgtaaatatttttgtgtaaacatccagtgc

R35(j8)L35

5’-gcactggatgtttacacaaaaatatttacactgccttttttttgagattgtaaaatattttatgtaaacagtgcactg

5’-cagtgcactgtttacataaaatattttacaatctcaaaaaaaaggcagtgtaaatatttttgtgtaaacatccagtgc

R35(j10)L35

5’-gcactggatgtttacacaaaaatatttacactgcctttttttttttgagattgtaaaatattttatgtaaacagtgcactg

5’-cagtgcactgtttacataaaatattttacaatctcaaaaaaaaaaggcagtgtaaatatttttgtgtaaacatccagtgc

L-TIR26 labeled

5’-gagattgtaaaatattttatgtaaac

5’FAM-gtttacataaaatattttacaatctc

R35r41 labeled

5’FAM-gcactggatgtttacacaaaaatatttacactgcctttttttacctagtcatcaatgcctacgtacgtaacgttac

5’-gtaacgttacgtacgtaggcattgatgactaggtaaaaaaaggcagtgtaaatatttttgtgtaaacatccagtgc

R35(j6)L35 labeled

5’FAM-gcactggatgtttacacaaaaatatttacactgccttttttgagattgtaaaatattttatgtaaacagtgcactg

5’-cagtgcactgtttacataaaatattttacaatctcaaaaaaggcagtgtaaatatttttgtgtaaacatccagtgc

R3541 double labeled

5’Cy5-gcactggatgtttacacaaaaatatttacactgcctttttttacctagtcatcaatgcctacgtacgtaacgttac

5’FAM-gtaacgttacgtacgtaggcattgatgactaggtaaaaaaaggcagtgtaaatatttttgtgtaaacatccagtgc

R35(j6)L35 double labeled

5’FAM-gcactggatgtttacacaaaaatatttacactgccttttttgagattgtaaaatattttatgtaaacagtgcactg

5’Cy5-cagtgcactgtttacataaaatattttacaatctcaaaaaaggcagtgtaaatatttttgtgtaaacatccagtgc

R35(j6)L35nick-3’OH double labeled

5’FAM-gcactggatgtttacacaaaaatatttacactgccttttttgagattgtaaaatattttatgtaaacagtgcactg

5’Cy5-cagtgcactgtttacataaaatattttacaatctc

5’-aaaaaaggcagtgtaaatatttttgtgtaaacatccagtgc

R35(j6)L35nick-ddC double labeled

5’FAM-gcactggatgtttacacaaaaatatttacactgccttttttgagattgtaaaatattttatgtaaacagtgcactg

5’Cy5-cagtgcactgtttacataaaatattttacaatctc(dd)

5’-aaaaaaggcagtgtaaatatttttgtgtaaacatccagtgc

R35(j6)L35 nick-5’OH double labeled

5’FAM-gcactggatgtttacacaaaaatatttacactgcctttttt

5’Cy5-cagtgcactgtttacataaaatattttacaatctcaaaaaaggcagtgtaaatatttttgtgtaaacatccagtgc

5’-gagattgtaaaatattttatgtaaacagtgcactg

R35(j6)L35-5’rec double labeled

5’FAM-gcactggatgtttacacaaaaatatttacactgcctttttt

5’Cy5-cagtgcactgtttacataaaatattttacaatctcaaaaaaggcagtgtaaatatttttgtgtaaacatccagtgc

5’-agattgtaaaatattttatgtaaacagtgcactg

R35(j6)L35-5’rec

5’-gcactggatgtttacacaaaaatatttacactgcctttttt

5’-cagtgcactgtttacataaaatattttacaatctcaaaaaaggcagtgtaaatatttttgtgtaaacatccagtgc

5’-agattgtaaaatattttatgtaaacagtgcactg

fR-TIR26

5’-tttttgggcagtgtaaatatttttgtgtaaac

5’-gtttacacaaaaatatttacactgcccaaaaa

R35(j6)L35 (6xT/A)

5’-gcactggatgtttacacaaaaatatttacactgccttttttgagattgtaaaatattttatgtaaacagtgcactg

5’-cagtgcactgtttacataaaatattttacaatctcaaaaaaggcagtgtaaatatttttgtgtaaacatccagtgc

R35(j6)L35 (6xG/C)

5’-gcactggatgtttacacaaaaatatttacactgccgggggggagattgtaaaatattttatgtaaacagtgcactg

5’-cagtgcactgtttacataaaatattttacaatctccccccccggcagtgtaaatatttttgtgtaaacatccagtgc

R35(j6)r14L12

5’-gcactggatgtttacacaaaaatatttacactgccttttttacagtaccatgctattttatgtaaacagtgcactg

5’-cagtgcactgtttacataaaatagcatggtactgtaaaaaaggcagtgtaaatatttttgtgtaaacatccagtgc

R35(j6)L14r21

5’-gcactggatgtttacacaaaaatatttacactgccttttttgagattgtaaaatagaccactggcagagtgcactg

5’-cagtgcactctgccagtggtctattttacaatctcaaaaaaggcagtgtaaatatttttgtgtaaacatccagtgc

R26(j6)L14

5’-gtttacacaaaaatatttacactgccttttttgagattgtaaaata

5’-tattttacaatctcaaaaaaggcagtgtaaatatttttgtgtaaac

R26(j6)L15

5’-gtttacacaaaaatatttacactgccttttttgagattgtaaaatat

5’-atattttacaatctcaaaaaaggcagtgtaaatatttttgtgtaaac

R26(j6)L14 double labeled

5’FAM-gtttacacaaaaatatttacactgccttttttgagattgtaaaata

5’Cy5-tattttacaatctcaaaaaaggcagtgtaaatatttttgtgtaaac

R26(j6)L14nick-3’OH double labeled

5’FAM-gtttacacaaaaatatttacactgcc

5’-ttttttgagattgtaaaata

5’Cy5-tattttacaatctcaaaaaaggcagtgtaaatatttttgtgtaaac

R26(j6)L14nick-ddC

5’FAM-gtttacacaaaaatatttacactgcc(dd)

5’-ttttttgagattgtaaaata

5’Cy5-tattttacaatctcaaaaaaggcagtgtaaatatttttgtgtaaac

R26(j6)L14nick-5’OH double labeled

5’FAM-gtttacacaaaaatatttacactgccttttttgagattgtaaaata

5’Cy5-tattttacaatctcaaaaaa

5’-ggcagtgtaaatatttttgtgtaaac

R26(j6)L14 5’rec double labeled

5’FAM-gtttacacaaaaatatttacactgcctttttttgagattgtaaaata

5’Cy5-tattttacaatctcaaaaaa

5’-gcagtgtaaatatttttgtgtaaac

R26(j6)L15 5’rec for crystallization

5’-gtttacacaaaaatatttacactgccgttttggagattgtaaaatat

5’-atattttacaatctccaaaac

5’-gcagtgtaaatatttttgtgtaaac

DNA fragments (gBlocks) used in the study (IDT)

gBlock 1

5’-

atgccgtaccatgggcgataaaattattcacctgactgacgacagttttgacacggatgtactcaaagcggacggggcgatcctcgtcg
atttctgggcagagtggtgcggtccgtgcaaaatgatcgccccgattctggatgaaatcgctgacgaatatcagggcaaactgaccgtt
gcaaaactgaacatcgatcaaaaccctggcactgcgccgaaatatggcatccgtggtatcccgactctgctgctgttcaaaaacggtga
agtggcggcaaccaaagtgggtgcactgtctaaaggtcagttgaaagagttcctcgacgctaacctggccggttctggttctggccata
tgcaccatcatcatcatcattctgagaatctgtactttcagggttctgctatggcacgtaagcgtatcatcactccagagaagaaggag
ctgatccgtaacctgattagcgaatacaacatcaccagcgctaaggatctgcaagaagcactgaaggatctgctgggtgatactatcca
aaacatgctggaggcagaactggatgaacatctgggttatgagaagtacgagtctactgaggaagcaaagtctaactaccgtaacggtt
acacctctaagaccctgaaatccagcgtaggtcaggtagaaatcgatatcccacgtgatcgtaacgctgaattcgagcctaaaatcgtc
ccgcgttacaaacgtgatatctccgagatcgagaacaaaatcatcgccatgtatgcgcgtggtatgtctactcgtgaaatcaacgaaca
gatccaggaaatctacggtttcgaagtcagcgcagaaatggtgtctaaaatcaccgacaaaatcctgccggaaatcgaagaatggcaga
aacgcccgctgggtgaagtttacccgattgttttcatcgatgcaatccacttctctgtcaaaaacgacggcatcgtaggtaaaaaagct
gtgtacatcgtgctggctatcgacatcgaaggtcagaaagacgtgatcggtatctacgtgggcgaaaacgaatcttctaaattctggct
gtccgtgctgaacgatctgaaaaaccgcggcgttaaagatatcctgatcctgtgcgctgacgctctgtctggcattaaagacgcgatta
atgcggcgtttccgaatactgaatatcagcgttgtatcgtacaccagattcgtaacacgctgaaatacgtttccgacaaagaccgtaaa
gaattcgcccgtgacctgaaacgtatttacaccgccccgaatgaaaaagcgggctatgaccagatgctggaagtttctgaaaaatggga
aaaaaaatacccggcggcgatgaaatcctggaaaagcaactgggacgttatttgcccgttcttcaaatacagcgaagaactgcgcaaaa
ttatgtacaccaccaacaccattgaaagcctgaactccagctatcgccgcattaacaaatcccgcacggtttttccgggcgaccagtct
ctgctgaaatccatttatctggccaccgttaaaattacgtccaaatggaccatgcgctataaaaattggggcctgattctgggccagct
gcagattatgtttgaaggccgcatttaataactcgagatcctga

gBlock 2

5’–

atgccgtaccatgggcgataaaattattcacctgactgacgacagttttgacacggatgtactcaaagcggacggggcgatcctcgtcg
atttctgggcagagtggtgcggtccgtgcaaaatgatcgccccgattctggatgaaatcgctgacgaatatcagggcaaactgaccgtt
gcaaaactgaacatcgatcaaaaccctggcactgcgccgaaatatggcatccgtggtatcccgactctgctgctgttcaaaaacggtga
agtggcggcaaccaaagtgggtgcactgtctaaaggtcagttgaaagagttcctcgacgctaacctggccggttctggttctggccata
tgcaccatcatcatcatcattctgagaatctgtactttcagggttctgctatggcacgtaagcgtatcatcactccagagaagaaggag
ctgatccgtaacctgattagcgaatacaacatcaccagcgctaaggatctgcaagaagcactgaaggatctgctgggtgatactatcca
aaacatgctggaggcagaactggatgaacatctgggttatgagaagtacgagtctactgaggaagcaaagtctaactaccgtaacggtt
acacctctaagaccctgaaatccagcgtaggtcaggtagaaatcgatatcccacgtgatcgtaacgctgaattcgagcctaaaatcgtc
ccgcgttacaaacgtgatatctccgagatcgagaacaaaatcatcgccatgtatgcgcgtggtatgtctactcgtgaaatcaacgaaca
gatccaggaaatctacggtttcgaagtcagcgcagaaatggtgtctaaaatcaccgacaaaatcctgccggaaatcgaagaatggcaga
aacgcccgctgggtgaagtttacccgattgttttcatcgctgcaatccacttctctgtcaaaaacgacggcatcgtaggtaaaaaagct
gtgtacatcgtgctggctatcgacatcgaaggtcagaaagacgtgatcggtatctacgtgggcgaaaacgaatcttctaaattctggct
gtccgtgctgaacgatctgaaaaaccgcggcgttaaagatatcctgatcctgtgcgctgacgctctgtctggcattaaagacgcgatta
atgcggcgtttccgaatactgaatatcagcgttgtatcgtacaccagattcgtaacacgctgaaatacgtttccgacaaagaccgtaaa
gaattcgcccgtgacctgaaacgtatttacaccgccccgaatgaaaaagcgggctatgaccagatgctggaagtttctgaaaaatggga
aaaaaaatacccggcggcgatgaaatcctggaaaagcaactgggacgttatttgcccgttcttcaaatacagcgaagaactgcgcaaaa
ttatgtacaccaccaacaccattgaaagcctgaactccagctatcgccgcattaacaaatcccgcacggtttttccgggcgaccagtct
ctgctgaaatccatttatctggccaccgttaaaattacgtccaaatggaccatgcgctataaaaattggggcctgattctgggccagct
gcagattatgtttgaaggccgcatttaataactcgagatcctga

gBlock 3

5’–

agagtgcaccatatgacagttccgttactttttggagattgtaaaatattttatgtaaactaattgactccttctatgatagaagccc
gcctgatgaatgctcatccaagcttggcgtaatcca
