# Supplementary material for: Structures of ISC th4 transpososomes reveal the role of asymmetry in copy‐out/paste‐in DNA transposition
Source: EMBO J. 2020 Oct 2;40(1):e105666. doi: 10.15252/embj.2020105666 (PMC7780238; doi:10.15252/embj.2020105666)
Supplement: Supplementary file 2 — Movie EV1 [file EMBJ-40-e105666-s002.zip › Legend_Movie_EV1.rtf]

Movie EV1 - Placement of transposon end into the active site of TnpAIn the pre-reaction complex (PRC), the flanking DNA (in magenta) keeps the 3’ terminal nucleotide of TIR (in red) out of the active site (residues D175, D241 and E348 showed as sticks). In the absence of flanking sequence, the 3’ terminus may enters the active site of the catalytic domain as approximated in pre-cleaved complex (PCC). This could reflect a transient motion when flanking sequence is bent under the flexible insertion domain to reach the active site. This step might involve DNA melting or deformation as observed in RAG1 complex (Ru et al, 2015; Chen et al, 2020). The color scheme is indicated in Fig 4D. 
