# Supplementary material for: Structures of ISC th4 transpososomes reveal the role of asymmetry in copy‐out/paste‐in DNA transposition
Source: EMBO J. 2020 Oct 2;40(1):e105666. doi: 10.15252/embj.2020105666 (PMC7780238; doi:10.15252/embj.2020105666)
Supplement: Supplementary file 3 — Movie EV2 [file EMBJ-40-e105666-s003.zip › Legend_Movie_EV2.rtf]

Movie EV2 - Generation of additional DNA binding site in STC1 In the pre-cleaved complex (PCC), the binding site for the spacer DNA (orange) and recipient TIR that we observe in strand transfer complex 1 (STC1) is not yet assembled. To generate a second DNA binding site (cdB), the catalytic domain must undergo a rigid body shift resulting in interlocked insertion domains. The 3’ terminal nucleotide of donor TIR is in red. The color scheme is indicated in Fig 4D.
