# Supplementary material for: Structures of ISC th4 transpososomes reveal the role of asymmetry in copy‐out/paste‐in DNA transposition
Source: EMBO J. 2020 Oct 2;40(1):e105666. doi: 10.15252/embj.2020105666 (PMC7780238; doi:10.15252/embj.2020105666)
Supplement: Supplementary file 4 — Source Data for Appendix [file EMBJ-40-e105666-s007.zip › EMBOJ-2020-105666R-Appendix_Figure_Source_Data-sd.pdf]

Source data for Fig S1B

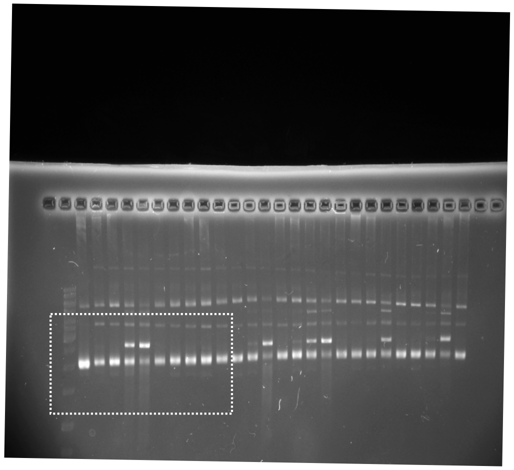

Source data for Fig S1C

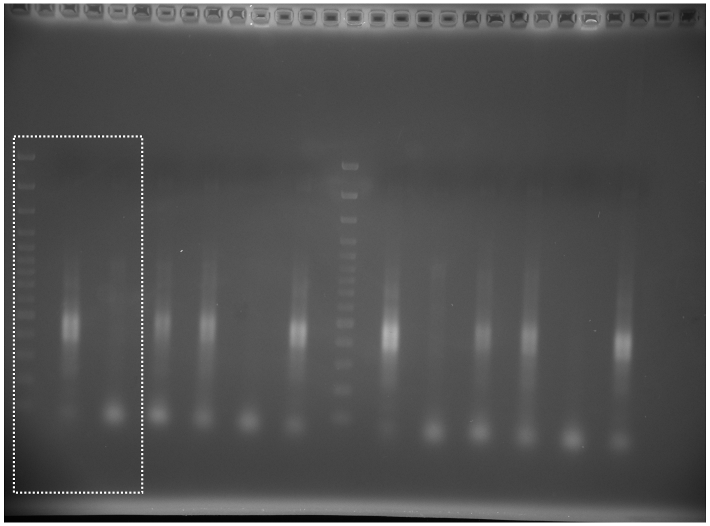

Source data for Fig S1D

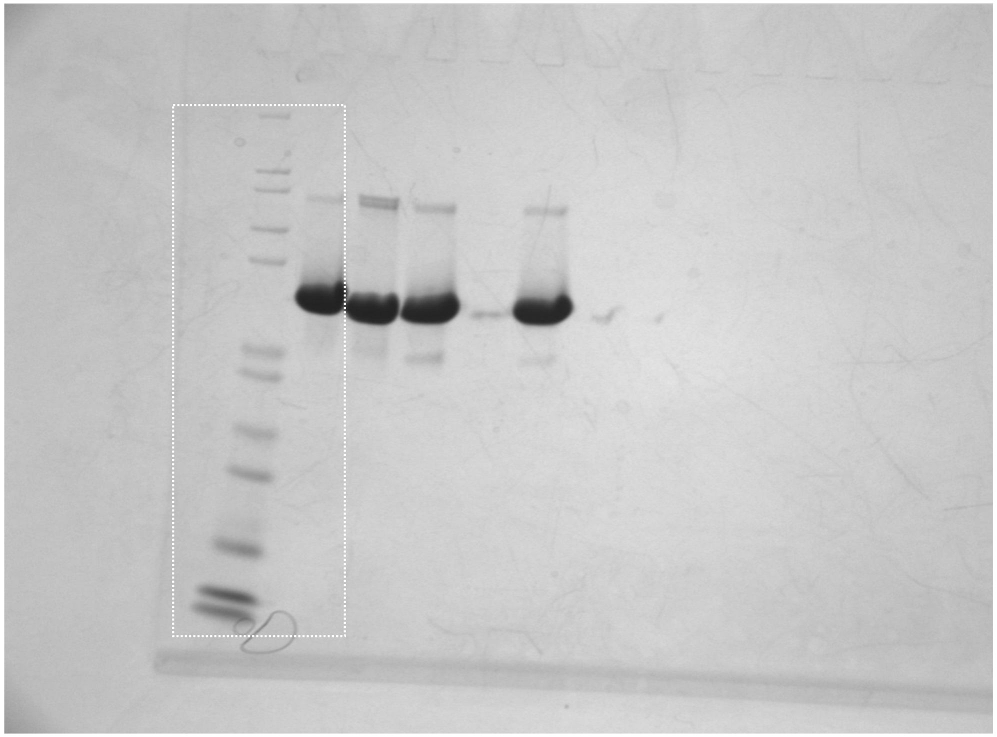

Fig S6

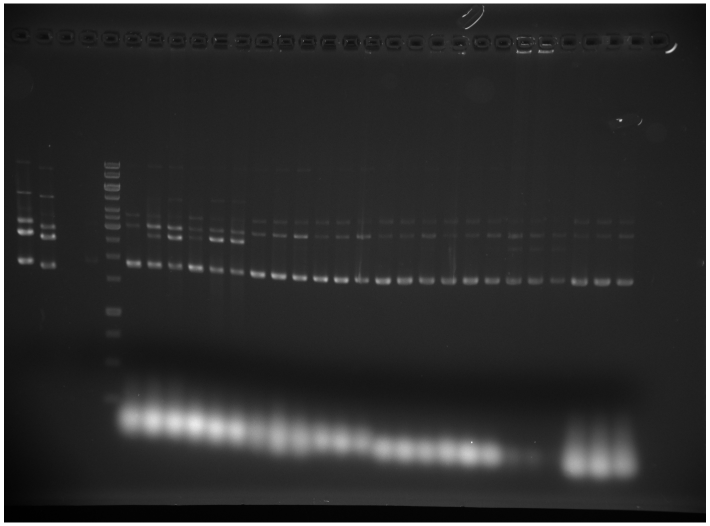

EtBr staining

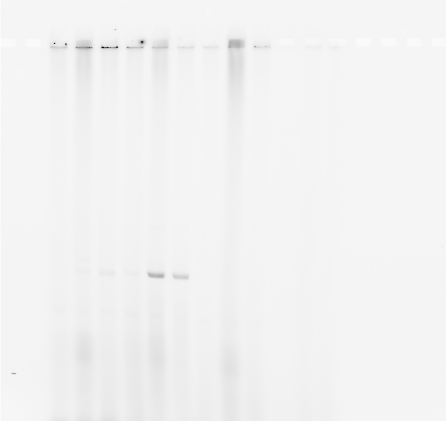

FAM fluorescent signal

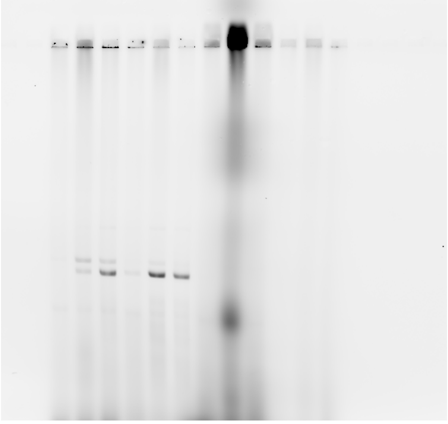

Cy5 fluorescent signal
