# Supplementary material for: Structures of ISC th4 transpososomes reveal the role of asymmetry in copy‐out/paste‐in DNA transposition
Source: EMBO J. 2020 Oct 2;40(1):e105666. doi: 10.15252/embj.2020105666 (PMC7780238; doi:10.15252/embj.2020105666)
Supplement: Supplementary file 6 — Source Data for Figure 2 [file EMBJ-40-e105666-s004.pdf]

Source data for Fig 2B

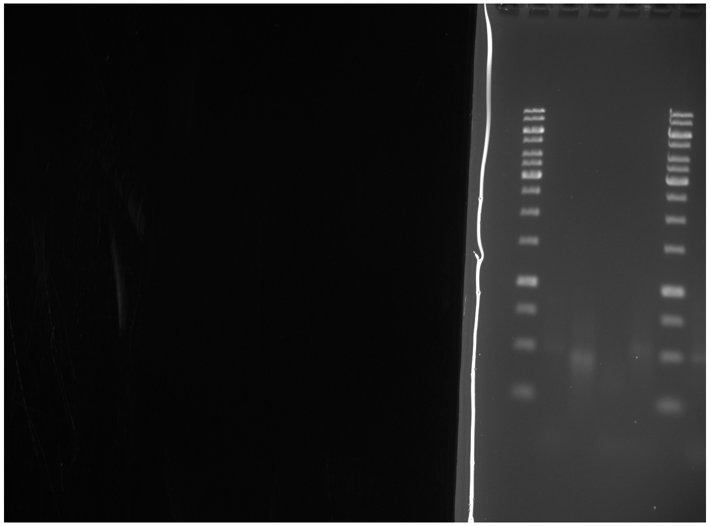

Source data for Fig 2E

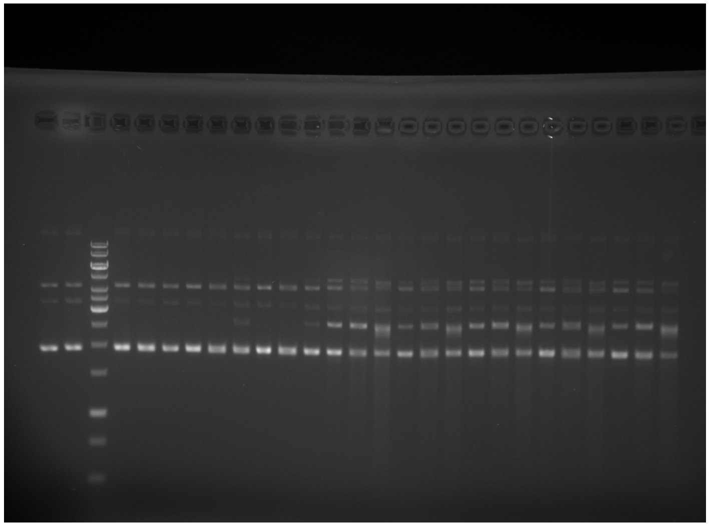

Source data for Fig 2F

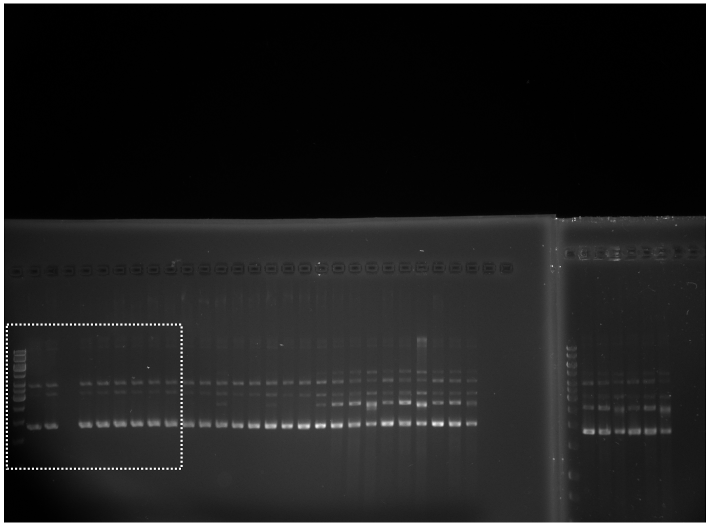

Source data for Fig 2G

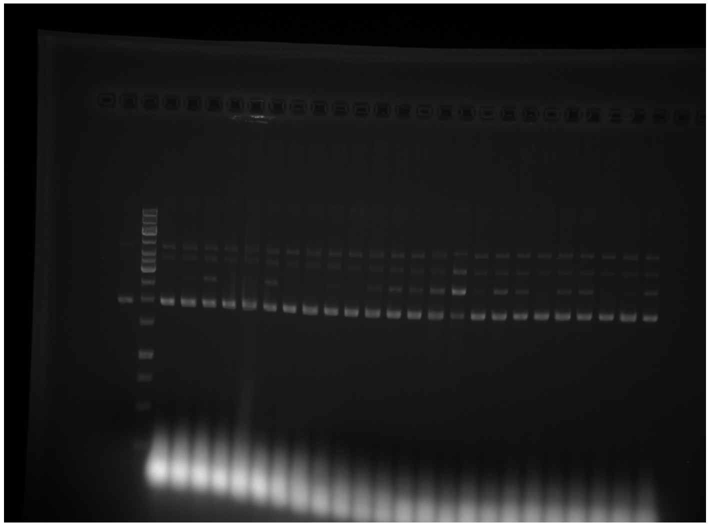

Source data for Fig 2H

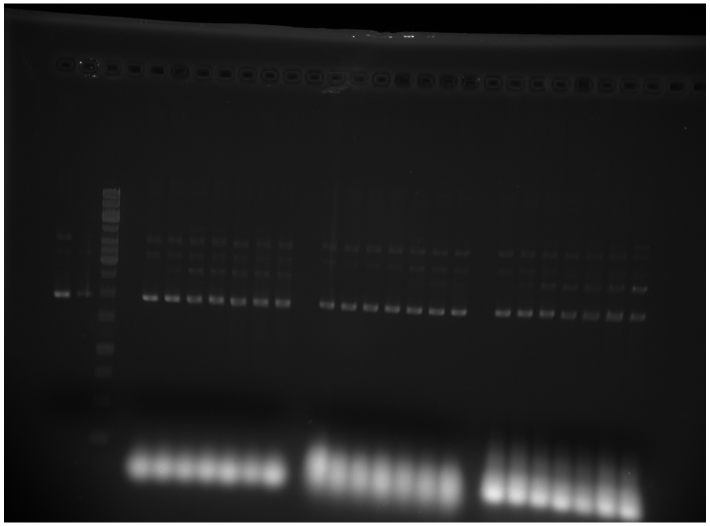
EtBr staining

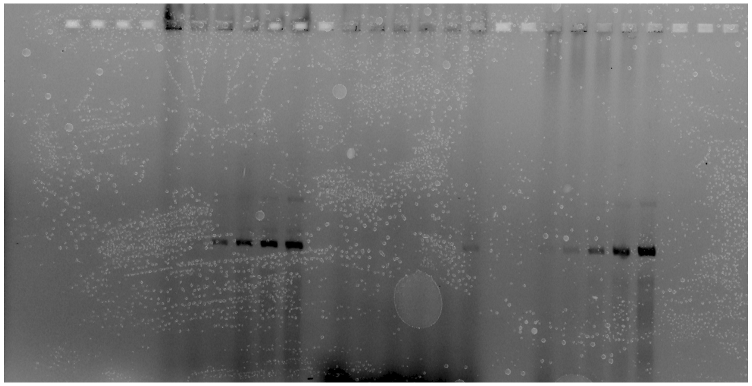
FAM fluorescent signal
