# Supplementary material for: Structures of ISC th4 transpososomes reveal the role of asymmetry in copy‐out/paste‐in DNA transposition
Source: EMBO J. 2020 Oct 2;40(1):e105666. doi: 10.15252/embj.2020105666 (PMC7780238; doi:10.15252/embj.2020105666)
Supplement: Supplementary file 7 — Source Data for Figure 3 [file EMBJ-40-e105666-s005.pdf]

Source data for Fig 3A

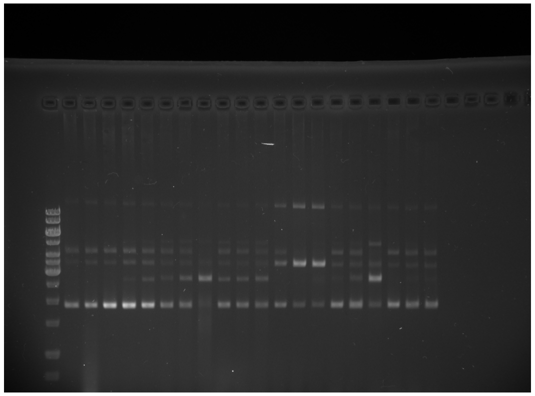

EtBr staining

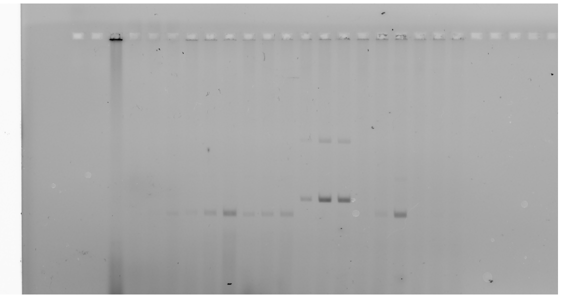

FAM fluorescent signal

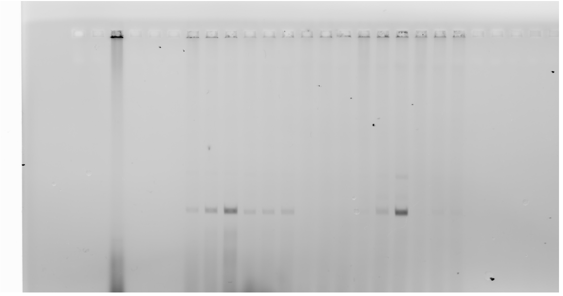

Cy5 fluorescent signal

Source data for Fig 3B

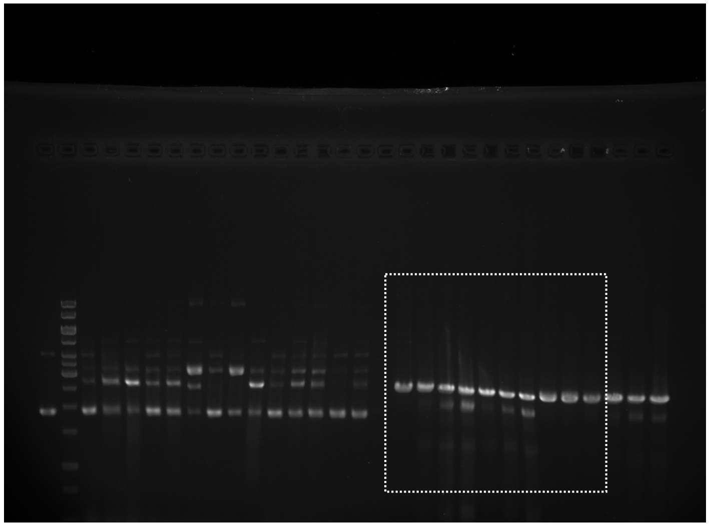

EtBr staining

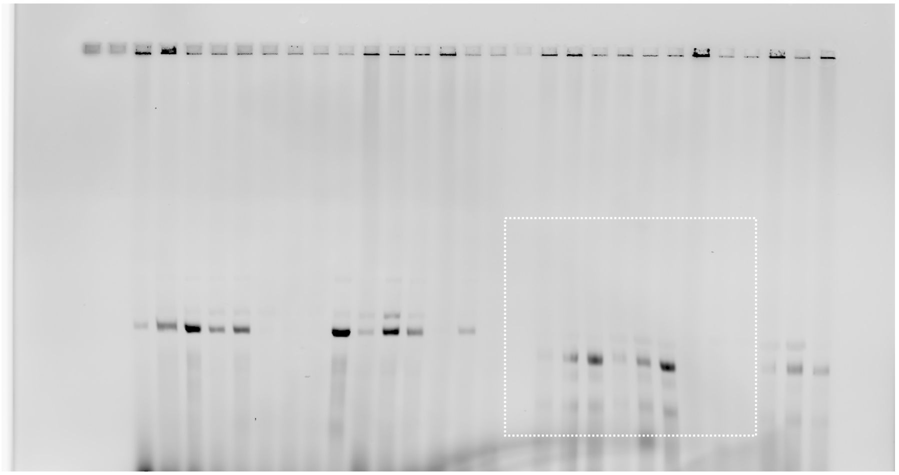

Cy5 fluorescent signal

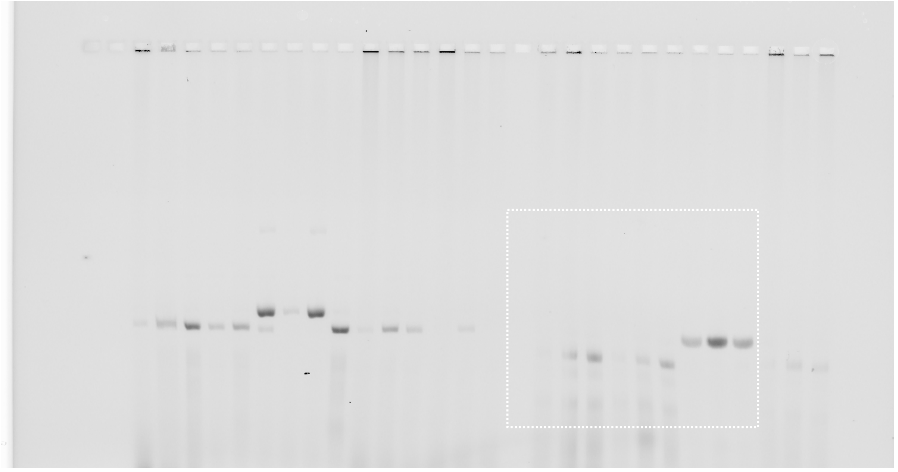

FAM fluorescent signal
