# Supplementary figures and images for: Structures of ISC th4 transpososomes reveal the role of asymmetry in copy‐out/paste‐in DNA transposition
Source: EMBO J. 2020 Oct 2;40(1):e105666. doi: 10.15252/embj.2020105666 (PMC7780238; doi:10.15252/embj.2020105666)

Source data for Fig 6G

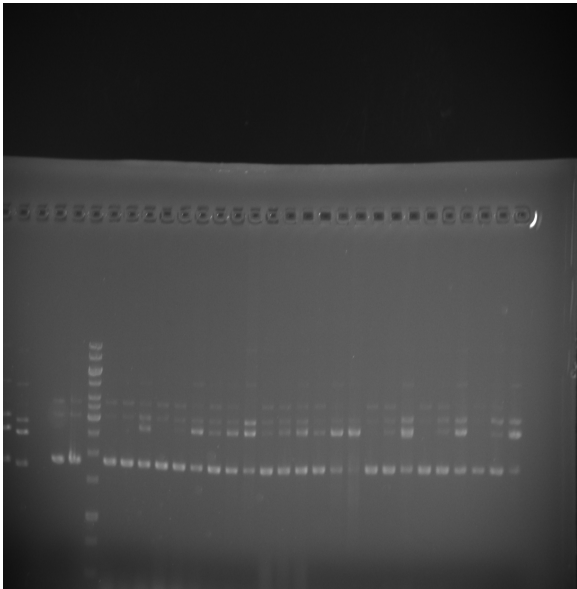

Supplement: Supplementary file 8 — Source Data for Figure 6 [file EMBJ-40-e105666-s006.pdf]
